# Supplementary material for: Mortality and its predictors among patients treated for acute exacerbations of chronic obstructive respiratory diseases in Jimma Medical Center; Jimma, Ethiopia: Prospective observational study
Source: PLoS One. 2020 Sep 23;15(9):e0239055. doi: 10.1371/journal.pone.0239055 (PMC7510970; doi:10.1371/journal.pone.0239055)
Supplement: S1 Annex — (DOCX) [file pone.0239055.s001.docx]

**S1 Annex**: Description of in-patient medications for the management of Asthma and COPD exacerbations.

| Medications | No of patients(N=130) | | In-patient medication dose and duration | |
| --- | --- | --- | --- | --- |
|  | Asthma(N=59) | COPD(N=71) |  |  |
|  |  |  | Asthma | COPD |
| **Antibiotics** | | | | |
| Ceftriaxone | 42 | 55 | 1gm bid for 5-7days | 1gm bid for 7-10 days |
| Azithromycin | 40 | 51 | 500mg /day for 5 days | 500mg /day for 5 days 5 days |
| Doxycycline | - | 3 | - | 100mg bid fro 5-7 days |
| Vancomycin | 1 | 4 | 1gm/ml bid for 5-7 days | 1gm/ml bid for 7-10 days |
| **Short acting b-agonsts and corticosteroids** | | | | |
| Salbutamol puff | 58 | 70 | 200mcg as needed | 200mcg as needed |
| Selmetrol puff | 2 | 3 | 50mcg bid | 50mcg bid for 1 month-2month |
| Bechlomethasone puff | 22 | 35 | 200mcg bid for 15 days-1month | 200mcg bid for 1-2months then reduce dose to 100mcg daily |
| Prednisolone tablet | 35 | 46 | 20-30mg for 5- 7 days | 40-60mg for 7-14 days |
| Hydrocortisone injections | 21 | 22 | 100-200mg/m, 1-3 doses for one days | 100-200mg/ml 1-3 doses for one days |
| Budesonide +formeterol inhalations | - | 5 | - | 160mcg+4.5mcg 1-2 month bid |
| **Oxygeen therapy** | | | | |
| Oxygen therapy | 40 | 54 | 6hrs-24hrs | 6hrs-24hrs ( may continue indefinitely) |

COPD: Chronic obstructive pulmonary disease
