# Supplementary material for: Mortality and its predictors among patients treated for acute exacerbations of chronic obstructive respiratory diseases in Jimma Medical Center; Jimma, Ethiopia: Prospective observational study
Source: PLoS One. 2020 Sep 23;15(9):e0239055. doi: 10.1371/journal.pone.0239055 (PMC7510970; doi:10.1371/journal.pone.0239055)
Supplement: S3 File — (PDF) [file pone.0239055.s004.pdf]

```
                                          ___  ____  ____  ____  ____(R)
                                         /__    /   ____/   /   ____/
                                        ___/   /   /___/   /   /___/
                                          Statistics/Data Analysis

                                            User: z
                                         Project: m
```

```
  ___  ____  ____  ____  ____ (R)
 /__    /   ____/   /   ____/
___/   /   /___/   /   /___/   14.0   Copyright 1985-2015 StataCorp LP
  Statistics/Data Analysis            StataCorp
                                      4905 Lakeway Drive
     MP - Parallel Edition            College Station, Texas 77845 USA
                                      800-STATA-PC        http://www.stata.com
                                      979-696-4600        stata@stata.com
                                      979-696-4601 (fax)

Single-user 8-core Stata perpetual license:
       Serial number:  10699393
         Licensed to:  Teshale Ayele
                       Mizan-Tepi university

Notes:
      1.  Unicode is supported; see help unicode_advice.
      2.  Maximum number of variables is set to 5000; see help set_maxvar.

1 . use "C:\Users\user\Desktop\THESIS\3 final3\epidat-stata\xy edited.dta", clear
(Dataform 1)

2 . stset length_hospital_stay, id(patient_code) failure(all_mortality==1) scale(1)

                id:  patient_code
     failure event:  all_mortality == 1
obs. time interval:  (length_hospital_stay[_n-1], length_hospital_stay]
 exit on or before:  failure

------------------------------------------------------------------------------
        130  total observations
          0  exclusions
------------------------------------------------------------------------------
        130  observations remaining, representing
        130  subjects
         14  failures in single-failure-per-subject data
       1412  total analysis time at risk and under observation
                                                at risk from t =         0
                                     earliest observed entry t =         0
                                          last observed exit t =        59

3 . stcox age ib(2).BMI_edited ib(2).adherence ib(1).duration_on_oxygen ib(2).bechlomethasone_inhala
> Frequency_Night_attacks ib(2).daily_activities

         failure _d:  all_mortality == 1
   analysis time _t:  length_hospital_stay
                 id:  patient_code

Iteration 0:   log likelihood = -41.989699
Iteration 1:   log likelihood = -30.355365
Iteration 2:   log likelihood = -29.919414
Iteration 3:   log likelihood = -29.894827
Iteration 4:   log likelihood = -29.894645
Iteration 5:   log likelihood = -29.894645
Refining estimates:
Iteration 0:   log likelihood = -29.894645

Cox regression -- no ties

No. of subjects =           84                  Number of obs    =         84
No. of failures =           14
Time at risk    =         1077
                                                LR chi2(8)       =      24.19
Log likelihood  =   -29.894645                  Prob > chi2      =     0.0021
```

| _t | Haz. Ratio | Std. Err. | z | P>\|z\| | [95% Conf. | Interval] |
|---|---|---|---|---|---|---|
| age | **1.065778** | **.0346167** | **1.96** | **0.050** | **1.000045** | **1.135832** |
| BMI_edited | | | | | | |
| <18.5kg/m2 | **2.213484** | **2.721195** | **0.65** | **0.518** | **.1988993** | **24.63314** |
| adherence | | | | | | |
| yes | **.8365846** | **.6751565** | **-0.22** | **0.825** | **.1720113** | **4.068767** |
| duration_on_oxygen | | | | | | |
| =>16hrs (prolonged) | **6.832947** | **5.784164** | **2.27** | **0.023** | **1.300339** | **35.90538** |
| bechlomethasone_inhalation | | | | | | |
| yes | **.8938572** | **1.136161** | **-0.09** | **0.930** | **.0740161** | **10.79469** |
| hydrocortisone_injections | | | | | | |
| yes | **2.956388** | **2.111105** | **1.52** | **0.129** | **.7293456** | **11.98366** |
| Frequency_Night_attacks | | | | | | |
| 7*/week | **.2409885** | **.2873097** | **-1.19** | **0.233** | **.0232907** | **2.493506** |
| daily_activities | | | | | | |
| yes | **3.391203** | **3.081058** | **1.34** | **0.179** | **.571478** | **20.12371** |

```
4 . save "C:\Users\user\Desktop\zzz2.dta"
  file C:\Users\user\Desktop\zzz2.dta saved

5 .
```
