# Supplementary material for: Mortality and its predictors among patients treated for acute exacerbations of chronic obstructive respiratory diseases in Jimma Medical Center; Jimma, Ethiopia: Prospective observational study
Source: PLoS One. 2020 Sep 23;15(9):e0239055. doi: 10.1371/journal.pone.0239055 (PMC7510970; doi:10.1371/journal.pone.0239055)
Supplement: Attachment — Submitted filename: Rebutal letter.docx [file pone.0239055.s005.docx]

**Rebuttal letter**

Dear editor, this is a rebuttal letter concerning the manuscript titled
“*Mortality and its predictors among patients admitted with acute exacerbation of chronic obstructive respiratory diseases in Jimma Medical Centre: Prospective observational study*” which you have requested for a response to the issues raised during the review process. Based on the comments given, all the authors have discussed on it and we have made a significant change to the manuscript, including the title (page 1). So, this is the highlight of what has been done. To make things easy, we kept all the concerns you raised unedited and provided our responses following each question.

1. Question 1 is about the format,

**Response**: it was considered. (Page 1 to the final)

2. Thank you for including your ethics statement:  The full protocol of this study was submitted for Institutional Review Board (IRB: IHRPGD/549/109) of JUMC, for ethical approval. After informing the overall concern of the study and confidentiality, written informed consent was taken written letter sheets; for those patients with problem of speaking, consent was requested from relatives.

Response: This section is re-written and included under the section of “**Study design, population and sample size determination**” on page 5 of the manuscript.

3. Please correct your reference to "p=0.000" to "p<0.001" or as similarly appropriate, as p values cannot equal zero.

 Response: We have corrected it

4. Please include additional information regarding the survey or questionnaire used in the study and ensure that you have provided sufficient details that others could replicate the analyses. For instance, if you developed a questionnaire as part of this study and it is not under a copyright more restrictive than CC-BY, please include a copy, in both the original language and English, as Supporting Information. Moreover, please include more details on how the questionnaire was pre-tested, and whether it was validated.

 Response: The questionnaire was sent to you and the data collection tool validation was provided under the sub-heading of “**Tool validation and data quality assurance”** on the page 7 of the manuscript.

5. We note that you have stated that you will provide repository information for your data at acceptance. Should your manuscript be accepted for publication, we will hold it until you provide the relevant accession numbers or DOIs necessary to access your data. If you wish to make changes to your Data Availability statement, please describe these changes in your cover letter and we will update your Data Availability statement to reflect the information you provide.

Response: Here I mean that the data will be sent to the journal if the manuscript is accepted for publication. So, you can directly access the data from the corresponding author if the manuscript is manuscript be accepted for publication.

6. Please amend either the abstract on the online submission form (via Edit Submission) or the abstract in the manuscript so that they are identical.

Response: Done

7. Your ethics statement must appear in the Methods section of your manuscript. If your ethics statement is written in any section besides the Methods, please move it to the Methods section and delete it from any other section. Please also ensure that your ethics statement is included in your manuscript, as the ethics section of your online submission will not be published alongside your manuscript.

Response: placed under method section on page 5 of the manuscript

**Reviewer's Responses to Questions**

Dear reviewers, your comments are fully accepted and we have addressed as follow
**Reviewer #1**: The manuscript, "Mortality and its predictors among patients admitted with acute exacerbations of chronic obstructive respiratory diseases in Jimma Medical Center: Prospective cohort study", by T. Mega and colleagues reports the outcomes of a cohort of patients admitted with COPD exacerbations to a referral hospital in a low-resource country. The study is descriptive, but still adds to our understanding of the outcomes for COPD patients in different countries. However, the study has several important limitations, which are outlined below:

1. There are several grammatical and spelling errors throughout the manuscript, which makes the manuscript difficult to read. For example, the third sentence of the fourth paragraph of the Background reads, "An indoor (like smoking), outdoor air pollution and occupational asthma gens were risks quantified for asthma in GBD". This sentence appears to be missing a word or phrase. Additionally, "gens" is a mistake, and the authors' intent is not clear regarding what word is meant to be in this sentence. The manuscript should be carefully reviewed and these errors corrected.

Response: Thank you for your concern indeed. All your comments are constructive and we reviewed the manuscript for the English grammar, sentence structures and punctuation. We consulted English language and literature department of Jimma University and included all the corrections they forward. For the word “asthmagens- any substance that **is** causally-related to the development of asthma symptoms” is a single word which was written as two separate words, “asthma gens.” We have corrected it in the manuscript.

2. The study population is very small given that the hospital serves a population of 15 million. The authors should provide reasons for this.

Response: Dear reviewer, we also acknowledge your concern regarding the sample size and that was why we have stated it as one of the major limitation. However, we would like to remind you that, this region had 4 additional referral hospitals and 20 general hospitals to which the cases can be admitted and managed. Jimma medical centre (a referral hospital with specialized care) is the highest level care in the region and it receives only patients referred from the aforementioned settings with the need of advanced medical care. So, it is likely that adequate sample size recruitment may need more than 6-months. Moreover, the authors didn’t get any funding support, one of hurdles for not extending the study period beyond six month. They covered all the data collection and investigational fees from their pocket. Because of this hardship, we were unable to prolong the study period.

3. The authors should provide specific criteria as to how the underlying diagnosis of asthma or COPD was made in the cohort patients.

Response: The comments is highly appreciated and we included this under the sub-heading of “Diagnostic procedures for acute exacerbations of chronic respiratory diseases” on the page 8 of the manuscript.
4. The authors should provide some context for the demographic information of their cohort. Specifically, they should indicate whether there are specific differences between the patients and the rest of the communities served by Jimma Hospital, or if the patient cohort is generally representative of the surrounding areas.

**Response**: As mentioned above, Jimma medical centre delivers the most advanced level of care and it served as a referral centre for selected paints. It has better diagnostic facility, human capacity and medication services. This is the final level of the health care system (tertiary care hospital) in Ethiopia. Cases are being managed here those who can’t be managed at the previously mentioned hospitals or those needing further investigation and case management.

5. There should be more details about the in-patient therapy received by the study cohort. Did any of the patients receive mechanical ventilation? What was the dose range and duration of steroid therapy used for treating the COPD/asthma exacerbations? This information will allow the interested reader to understand the differences in therapy received by the patients in the Jimma cohort and other COPD populations.

Response: Patients receiving mechanical ventilation were not included as they are not stable enough to provide lung function test. There were actually 3 patients receiving mechanical ventilation during this study and excluded. The details of the inpatient medication including corticosteroids for asthma and COPD were presented (Annex 1) .
6. The authors should summarize the post-hospital follow up provided to the patients. Was there a systematic post-discharge educational program given to the patients? Is there a community-based nursing follow up available?

Response: the highlight of the post-hospital care was provided under the sub-heading of “**Description of Medications and investigational findings (page 14)”** and there is no-community based nursing home. Hence, they will be linked to the OPD of JMC pulmonary clinic for regular follow-up.
7. The finding that patients with comorbid disease were less likely to die during follow up compared to patients without comorbidities is unexpected. The authors should enlarge their Discussion of this finding to include their assessment as to the reasons for this.

Response: we ask an apology for the fatal error we committed during the data analysis. As you saw this finding was contradictory not only with existing studies, but also with the descriptive section too. i.e 9 of the 14 deaths had comorbidity, and comorbidity become protective. With this controversial finding, thank you for your insight, we recalled the statistician to re-analyse the data and found that the variable “co-morbidity” should have been removed earlier as its binary cox regression p-value was 0.63. There are also other variables with similar fate. Thus, we revised this section and provided a new table with some modification. For the sake of convenience we have sent you the final model of the STATA output.

**Reviewer 2**

This is an interesting study and the objective of the study are well stated. In the introduction, the objectives are stated clearly: explore in-hospital of asthma and COPD exacerbations in low-income nations.
1) Title- while this study is from Jimma Ethiopia, the authors may want to re-consider their title and add Ethiopia after Jimma.

Response: Done!

2) Sample size- the authors chose a 6 month study period with a relatively small 'N" of 130, did the authors consider extending the study period to 12 months to capture more patients.

- Response: Dear reviewer, we also acknowledge your concern regarding the sample size and that was why we have stated it as one of the major limitation. However, we would like to remind you that, this region had 4 additional referral hospitals and 20 general hospitals to which the cases can be admitted and managed. Jimma medical centre (a referral hospital with specialized care) is the highest level care in the region and it receives only patients referred from the aforementioned settings with the need of advanced medical care. So, it is likely that adequate sample size recruitment may need more than 6-months. Moreover, the authors didn’t get any funding support, one of hurdles for not extending the study period beyond six month. They covered all the data collection and investigational fees from their pocket. Because of this hardship, we were unable to prolong the study period.
  3) Grammatical errors: There are numerous spelling, syntax and grammatical errors throughout the manuscript which I have noted below.

Response: Corrections made
3a) Abstract in methods: "Kaplan-Mayer (Log-rank test) and Cox regression" The crrect spelling is Kaplan Meier.

- Response: Corrected
  3b) Abstract: "The total proportion of in-hospital mortality was 14(10.78%), making the incidence rate of mortality, 2.56 per 1000 person-years; 95% CI [1.518-4.328]." The authors should re-word this sentence as it is unclear as written.

Response: Re-written
