# Supplementary material for: Mortality and its predictors among patients treated for acute exacerbations of chronic obstructive respiratory diseases in Jimma Medical Center; Jimma, Ethiopia: Prospective observational study
Source: PLoS One. 2020 Sep 23;15(9):e0239055. doi: 10.1371/journal.pone.0239055 (PMC7510970; doi:10.1371/journal.pone.0239055)
Supplement: Attachment — Submitted filename: Rebuttal letter.docx [file pone.0239055.s006.docx]

**Response to Reviewers'**

Dear reviewers, the authors will fully acknowledge the importance of your feedbacks to produce a scientifically sound manuscript. Therefore, we incorporated them to the manuscript given they are directly related to the objectives defined by the investigators. Other points intended for explanation were described on the rebuttal letter only. The comments were addressed in the following manner; keeping all statements of the reviewers as they were.

**Reviewer #1**:

The revised manuscript by **T. Mega** and colleagues has been much improved by the authors’ revisions. However, this study continues to have significant limitations, which are summarized below:

1. While improved, the writing continues to have many spelling, grammar, and word choice errors. Examples of these include:
a. Line 108 “cathment” instead of “catchment”
b. Line 120 “miss diagnosed” instead of “misdiagnosed”
c. Line 169 “throghly” instead of “thoroughly”
d. Line 169 “discudssed” instead of “discussed”
e. Line 234 “Moreover” is awkward.
f. Line 247 “peviuos” instead of “previous”
g. Line 258 “chronic comorbidities and it contributed” - the subject of “contributed” should be plural
h. Line 337 “predictor” instead of “predictors”
i. Line 393 “prevents” is not the correct word to describe an inverse association between comorbid disease and mortality.

The authors need to have the manuscript reviewed again in detail to correct these English errors

**Response:** The whole document was fully revised for these and other errors.

2. The description of the study population would be improved by providing additional context for the interested reader. For example, how does the high prevalence of smoking (21.5%) in the study population compare to the overall prevalence in Ethiopia?

**Response:** Though no recent data regarding the national prevalence of smoking, the 2015 published study by WHO indicated 8.9% and 0.5% of Ethiopian men and women, respectively, were smokers, describing it as an ongoing and direct public health threat. (<https://tobaccoatlas.org/wp-content/uploads/pdf/ethiopia-country-facts.pdf>). In the context of this manuscript, such high prevalence of smoking may not be surprising as majority of the study participants were COPD (71/130) patients and smoking is the well-known risk factor.

Approximately half of the subjects were from rural areas – does this reflect the overall population in the hospital catchment area?

**Response:** Yes! The rural population in Ethiopia was 78.78 % in 2019 (World Bank). So, this catchment area also mirrors similar trend.

Additionally, since a substantial percentage (56%) of the study subjects did not have formal education, what percentage of the general population in the hospital catchment area and/or Ethiopia similarly does not have formal education?

**Response:** The national literacy rate as reported by World Bank in 2017 was 52% (<https://data.worldbank.org/indicator/SE.ADT.LITR.ZS>). So, the data reflects similar fact.

3. The authors should provide additional information about the use of antibiotics for the patients, particularly since their use correlated with mortality. Since the subjects were all admitted with exacerbations of lung disease, the number of subjects who did not receive antibiotics is high. Are there hospital guidelines regarding the use of antibiotics? If not, is there a consensus among the practitioners in the hospital regarding the use of antibiotics for patients admitted with exacerbations of lung disease? Is the use of antibiotics likely to be a marker of disease severity? Why did the authors not include statistics regarding antibiotic use?

**Response:** Dear reviewer, it is a global fact that all patients admitted with acute exacerbations of asthma and COPD must receive a course of antibiotics. This data was annexed as inpatient medication in the table named “annex A” in our manuscript after table of contents. If you see the data regarding inpatient medications in this table, the number of patients exposed to antibiotics was far beyond the study population, as a patient might receive one or more antibiotics.

**Regarding exclusion of antibiotics data from statistics section**: the reviewers should note that not only antibiotics but also other several variables were removed because of the fact that either the variables were failed on cell adequacy test or they were removed from the model due to their p-value >0.25 on binary logistic regression analysis.

4. The Discussion still includes a statement (line 393) that the presence of comorbid disease was protective. However, the findings reported in the Results indicate that pulmonary hypertension, heart failure, and renal dysfunction were all associated with increased mortality. This discordance should be resolved.

**Response:** This expression was erroneous and corrected according to the finally corrected data. You can also look at the STATA output (the final model) attached as supplementary data in our previous revision sent to *PLOS ONe*.

5. The authors need to expand their discussion of the study limitations. The small sample size resulted in the study lacking statistical power. The study therefore likely missed predictors of mortality. An example of this would be the lack of association between prior hospitalization and mortality. The authors need to more fully outline this limitation of their study. Additionally, the exclusion of patients requiring mechanical ventilation needs to be described as a limitation. Although the authors provide the rationale for this exclusion, patients who require mechanical ventilation for an exacerbation of lung disease have a high in-hospital mortality. Thus, excluding this population limited the study’s ability to detect risk factors for in-hospital mortality.

**Response:** We included these precious statements in our limitation section. Thank you indeed!

6. Table 5 has an asterisk after the number of night attacks. It isn't clear if this refers to something that should have additional clarification in the Table legend or if this is a typographical error. The authors should either clarify the use of the asterisk or remove it.

**Response:** Dear reviewer, these are not asterisk, by to say “times” (sign of multiplication). Anyhow removing the asterisk will not affect the content of the information and so, we have removed them.
